# Few individuals with Lennox-Gastaut syndrome have autism spectrum disorder: a comparison with Dravet syndrome

**DOI:** 10.1186/s11689-018-9229-x

**Published:** 2018-03-20

**Authors:** Na He, Bing-Mei Li, Zhao-Xia Li, Jie Wang, Xiao-Rong Liu, Heng Meng, Bin Tang, Wen-Jun Bian, Yi-Wu Shi, Wei-Ping Liao

**Affiliations:** 1 Institute of Neuroscience and Department of Neurology of the Second Affiliated Hospital of Guangzhou Medical University, Chang-gang-dong Road 250, Guangzhou, 510260 China; 2 Key Laboratory of Neurogenetics and Channelopathies of Guangdong Province and the Ministry of Education of China, Guangzhou, 510260 China; 3 Department of Neurology, The First Affiliated Hospital of Jinan University, Guangdong, 510630 China; 4 Clinical Neuroscience Institute of Jinan University, Guangdong, 510630 China

**Keywords:** Autism spectrum disorder, Intellectual disability, Epileptic encephalopathy, Lennox-Gastaut syndrome, Dravet syndrome

## Abstract

**Background:**

Autism spectrum disorder (ASD) in epilepsy has been a topic of increasing interest, which in general occurs in 15–35% of the patients with epilepsy, more frequently in those with intellectual disability (ID). Lennox-Gastaut syndrome (LGS) and Dravet syndrome (DS) are two typical forms of intractable epileptic encephalopathy associated with ID. We previously reported that ASD was diagnosed in 24.3% of patients with DS, higher in those with profound ID. Given the severe epilepsy and high frequency of ID in LGS, it is necessary to know whether ASD is a common psychomotor co-morbidity of LGS. This study evaluated the autistic behaviors and intelligence in patients with LGS and further compared that between LGS and DS, aiming to understand the complex pathogenesis of epilepsy-ASD-ID triad.

**Methods:**

A total of 50 patients with LGS and 45 patients with DS were enrolled and followed up for at least 3 years. The clinical characteristics were analyzed, and evaluations of ASD and ID were performed.

**Results:**

No patients with LGS fully met the diagnostic criteria for ASD, but three of them exhibited more or less autistic behaviors. Majority (86%) of LGS patients presented ID, among which moderate to severe ID was the most common. Early onset age and symptomatic etiology were risk predictors for ID. The prevalence of ASD in LGS was significantly lower than that in DS (0/50 vs. 10/45, *p* < 0.001), while the prevalence and severity of ID showed no significant difference between the two forms of epileptic encephalopathy.

**Conclusions:**

This study demonstrated a significant difference in the co-morbidity of ASD between LGS and DS, although they had a similar prevalence and severity of ID, refuting the proposal that the prevalence of ASD in epilepsy is accounted for by ID. These findings suggest that the co-morbidity of ASD, ID, and epilepsy may result from multifaceted pathogenic mechanisms.

## Background

Lennox-Gastaut syndrome (LGS) is a severe epileptic encephalopathy, which accounts for approximately 1–10% of childhood epilepsies [1]. The etiologies of LGS can be symptomatic with an identifiable brain disorder, or cryptogenic without known causes [2]. The clinical presentation of LGS is characterized by the following triad: multiple seizure types that are mainly tonic, specific abnormal electroencephalogram (EEG), and cognitive impairment. Majority of LGS patients typically experience cognitive regression at seizure onset, with a decreasing intelligence quotient (IQ) over time [3]. Along with cognitive problems, behavioral and psychiatric co-morbidities are commonly seen in patients with LGS, such as hyperactivity, anxiety, aggression, and depression [2]. Autism or autistic behavior has also been reported, but only in a few cases of LGS [4, 5].

Autism spectrum disorder (ASD) is a complex neurobehavioral disorder characterized by social interaction impairments, communication deficits, and stereotyped behaviors. It has been estimated that ASD may occur in 15–35% of children with epilepsy, while epilepsy may affect 7–46% of patients with ASD [6], suggesting a strong relationship between ASD and epilepsy. Additionally, previous evidence indicates that ASD occurs more frequently in epilepsy patients with intellectual disability (ID) [7]. We previously reported that ASD was diagnosed in 24.3% of patients with Dravet syndrome (DS), which is another form of epileptic encephalopathy with ID, and ASD was more often in those with profound ID [8]. Given the severe epilepsy and high frequency of ID in LGS, it is necessary to know whether autism or autistic behavior is a common psychomotor co-morbidity of LGS. As little is known about the relative contributions of epilepsy itself, ID, or other underlying factors to the occurrence of ASD in different forms of epileptic encephalopathy, a comparison between LGS and DS will help to understand the complex pathogenesis of epilepsy-ASD-ID triad.

In this study, we evaluated the autistic behaviors and intelligence in patients with LGS and further compared that between LGS and DS, aiming to explore the prevalence of ASD and its potential risk factors.

## Methods

### Participants

A total of 50 patients with LGS and 45 patients with DS were recruited between 2007 and 2013 at the Epilepsy Centre of the Second Affiliated Hospital of Guangzhou Medical University. All patients were southern Han Chinese. Medical records were collected, including gender, seizure onset age, seizure types and frequency, application of antiepileptic drugs (AEDs), family history, brain MRI scans, and video-EEG records. This study adhered to the guidelines of the International Committee of Medical Journal Editors with regard to patient consent for research or participation, and study protocol was approved by the ethics committee of the hospital.

According to the criteria of Commission on Classification and Terminology of the International League Against Epilepsy [9–11], LGS was diagnosed when at least two of the following criteria were met: (1) multiple seizure types including tonic seizure, (2) generalized polyspikes or fast rhythms during sleep (required especially when daily tonic seizures are obscure), and (3) diffuse slow (≤ 2.5 Hz) spike-wave complex on EEG. Tonic seizures are essential for the diagnosis of LGS. The diagnosis of Dravet syndrome was based on the criteria (1) febrile seizures starting in the first year of life and subsequent appearance of multiple seizure types (myoclonic, atypical absence, focal); (2) prolonged seizures triggered by or sensitive to fever, which might evolve to status epilepticus; (3) generalized and focal/multifocal discharges on EEG; and (4) normal psychomotor development before onset of seizure, but cognitive regression afterward. All EEGs were reviewed by two qualified electroencephalographers. Epileptic seizures and epilepsy syndromes were diagnosed and classified by two epileptologists in our Epilepsy Center.

### Neurodevelopment assessments

ASD was diagnosed according to the Diagnostic and Statistical Manual of Mental Disorders, Fifth Edition (DSM-5), and the International Classification of Diseases, Tenth Edition (ICD-10). A diagnosis of ASD was made when patients had deficits in two core domains: (1) deficits in social communication and social interaction and (2) restricted repetitive patterns of behavior, interests, and activities. The Autism Behavior Checklist (ABC) by the parents and the Childhood Autism Rating Scale (CARS) by the same qualified psychiatrists were used as additional assessments. An ABC score of ≥ 67 and a CARS score of ≥ 30 were considered to be supporting the diagnosis. Autism Diagnostic Observation Schedule (ADOS), Autism Diagnostic Interview (ADI), and Diagnostic Interview for Social and Communication Disorders (DISCO) were occasionally used due to the lack of Chinese norms of these tools.

To assess cognitive impairment, Chinese Wechsler Intelligence Scale for Children (C-WISC), for those aged ≥ 6 years, and Gesell Developmental Scales, for those aged < 6 years, were performed by the same qualified psychiatrists in our hospital. According to IQ and developmental quotient (DQ), the cognitive outcomes were divided into five categories as in our previous study [8]: normal or borderline intelligence (IQ > 70 or DQ > 75), mild ID (IQ ranging from 55 to 70 or DQ ranging from 55 to 75), moderate ID (IQ/DQ ranging from 40 to 54), severe ID (IQ/DQ ranging from 25 to 39), and profound ID (IQ/DQ below 25).

### Statistical analysis

Student *t* tests and *χ*^2^ tests were performed to determine the significance of differences between groups. Two-sided *p*-values < 0.05 were considered statistically significant. All analysis was performed using SPSS version 19.0 (SPSS, Chicago, IL, USA).

## Results

### Demographic and clinical characteristics of patients with LGS

The 50 patients included 38 males and 12 females, aged from 2.2 to 33 years (mean age of 9.3 years, at cognitive evaluation), and were followed up for at least 3 years (range 3–6 years). Their clinical characteristics are summarized in Table 1.

**Table 1:** Demographic and clinical characteristics of patients with Lennox-Gastaut syndrome

	LGS (*N* = 50)	Cryptogenic LGS (*N* = 28)	Symptomatic LGS (*N* = 22)	*p*
Gender (male/female)	38/12	21/7	17/5	0.852
Age at conclusion of study (years)	12.9 ± 6.5	12.5 ± 6.1	13.5 ± 7.1	0.875
Age at evaluation (years)	9.3 ± 6.2	9.9 ± 5.6	8.4 ± 7.0	0.418
Age at seizure onset (months)	35.3 ± 29.6	49.4 ± 29.5	17.4 ± 18.1	0.007*
Seizure type				
Tonic	48 (96%)	26 (92.9%)	22 (100.0%)	0.309
GTCS/secondary GTCS	34 (68%)	18 (64.3%)	16 (72.7%)	0.525
Complex/simple partial	20 (40%)	11 (39.3%)	9 (40.9%)	0.907
Atypical absence	20 (40%)	12 (42.9%)	8 (36.4%)	0.642
Myoclonic	16 (32%)	8 (28.6%)	8 (36.4%)	0.558
Spasms	12 (24%)	1 (3.6%)	11 (50.0%)	0.001*
Drops	11 (22%)	5 (17.9%)	6 (27.3%)	0.650
Atonic	8 (16%)	5 (17.9%)	3 (13.6%)	0.988
Status epilepticus	6 (12%)	3 (10.7%)	3 (13.6%)	1.000
EEG characteristics				
Slow background activity	34 (68%)	18 (64.3%)	16 (72.7%)	0.525
Generalized polyspikes	49 (98%)	28 (100.0%)	21 (95.5%)	0.440
Diffuse SSW pattern	36 (72%)	22 (78.6%)	14 (63.6%)	0.243
Focal discharges	43 (86%)	24 (85.7%)	19 (86.4%)	1.000
Burst-suppression pattern	11 (22%)	6 (21.4%)	5 (22.7%)	1.000
Antiepileptic drugs				
2	13 (26%)	10 (35.7%)	3 (13.6%)	0.768
≥ 3	37 (74%)	18 (64.3%)	19 (86.4%)	
Seizure free	14 (28%)	13 (46.4%)	1 (4.5%)	0.001*

*GTCS* generalized tonic-clonic seizure, *SSW* slow spike-wave

^*^ *p* < 0.05 (two-sided) was statistically significant

The age at onset of seizure ranged from 10 days to 9 years, with the mean onset age of 35.3 months. Eight of the 50 (16%) patients evolved from infantile spasms. All patients experienced multiple seizure types, among which tonic seizure was the most common, followed by generalized tonic-clonic seizure (GTCS)/secondary GTCS. All patients were treated with 2–6 AEDs (mean 3.4). Fourteen patients (28%) were seizure-free for at least 1.5 year, and one patient has been seizure-free for more than 6 years under the combination of valproate and lamotrigine.

Based on brain MRI and medical history, 22 patients (44.0%) had known etiologies and were classified as symptomatic LGS. These etiologies included hypoxic-ischemic encephalopathy, intracranial hemorrhage, malformations of cortical development, ventriculomegaly, tuberous sclerosis, head trauma, encephalitis, hydrocephalus, and porencephaly. The other 28 patients (56.0%) had no identifiable etiology and were classified as cryptogenic LGS. Clinical characteristics of the two groups were compared (Table 1). Patients with symptomatic LGS had much earlier onset age (17.4 vs. 49.4 months in patients with cryptogenic LGS, *p* = 0.007). Spasms were more common in patients with symptomatic LGS than that in cryptogenic LGS patients (11/22 vs. 1/28, *p* < 0.001). The patients who were seizure-free were significantly less in the symptomatic LGS group than that in the cryptogenic LGS group (4.5 vs. 46.4%, *p* < 0.001).

### Autism and autistic behaviors in LGS

No patient with LGS could be diagnosed as ASD, and the average scores of ABC and CARS were 22.1 and 20.7, respectively.

However, three patients exhibited more or less autistic behaviors. The three patients showed speech delay and repetitive stereotypic movements. One of them had social interaction reduction, or narrow interests, or narrow interests and short temper each. Their etiologies were intracranial hemorrhage, encephalitis, and hypoxic-ischemic encephalopathy, respectively. One of them was evolved from infantile spasms. They also had a very early onset age, which were 2, 4, and 6 months, respectively. Their cognitive outcomes were poor, one patient was complicated with severe ID, and the other two had profound ID.

### Intellectual disability in LGS and the potential risk factors

C-WISC was performed on 44 patients, and Gesell Developmental Scale was performed on the other 6 patients. According to the IQ or DQ values, 7 patients (14%) had normal or borderline intelligence, and the majority (86%) presented different levels of ID, including 10 patients (20%) with mild ID, 4 (8%) with moderate ID, 19 (38%) with severe ID, and 10 (20%) with profound ID.

To explore the potential risk factors for ID in patients with LGS, clinical characteristics in patients with and without ID were compared (Table 2). The patients with ID had a much earlier onset age than those without ID (26.2 vs. 50.6 months, *p* = 0.009), suggesting that seizure onset age was potentially a significant predictor for ID. Half of the patients with ID had a symptomatic etiology, significantly more often than those without ID (22/43 vs. 0/7, *p* = 0.014), implying that symptomatic etiology was potentially another risk predictor.

**Table 2:** Comparison of clinical features among LGS patients with and without intellectual disability

	Without ID (*N* = 7)	With ID (*N* = 43)	*p*
Gender (male)	5 (71.4%)	33 (76.7%)	1.000
Age at seizure onset (month)	50.6 ± 35.6	26.2 ± 20.0	0.009*
Age at evaluation (years)	11.9 ± 8.6	8.9 ± 5.8	0.400
History of infantile spasms	0 (0.0%)	8 (18.6%)	0.580
Seizure types			
Tonic	6 (85.7%)	42 (97.7%)	0.263
GTCS/secondary GTCS	5 (71.4%)	29 (67.4%)	1.000
Complex partial seizure	2 (28.6%)	18 (41.9%)	0.687
Atypical absence	4 (57.1%)	16 (37.2%)	0.416
Myoclonic	2 (28.6%)	14 (32.6%)	1.000
Spasms	0 (0.0%)	12 (27.9%)	0.174
Drops	1 (14.3%)	10 (23.3%)	1.000
Atonic	1 (14.3%)	7 (16.3%)	1.000
Status epilepticus	1 (14.3%)	5 (11.6%)	1.000
EEG characteristics			
Slow background activity	4 (57.1%)	30 (69.8%)	0.666
Generalized polyspikes	7 (100.0%)	42 (97.7%)	1.000
Diffuse SSW pattern	6 (85.7%)	30 (69.8%)	0.657
Focal discharges	5 (71.4%)	38 (88.4%)	0.250
Burst-suppression pattern	0 (0.0%)	11 (25.6%)	0.324
Antiepileptic drugs			
2	3 (42.9%)	10 (23.3%)	0.357
≥ 3	4 (57.1%)	33 (76.7%)	
Etiology			
Symptomatic	0 (0.0%)	22 (51.2%)	0.014*
Cryptogenic	7 (100.0%)	21 (48.8%)	
Seizure free	4 (57.1%)	10 (23.3%)	0.085

*GTCS* generalized tonic-clonic seizure, *SSW* slow spike-wave

^*^ *p* < 0.05 (two-sided) was statistically significant

**Fig. 1:**
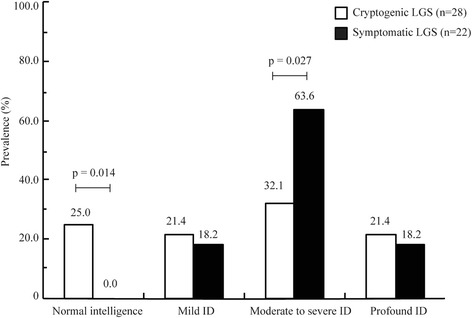
Comparison of ID in patients with cryptogenic LGS and those with symptomatic LGS. The cognitive outcome in patients with cryptogenic LGS was significantly better than that in patients with symptomatic LGS (*p* = 0.040). In the cryptogenic LGS group, the patients with normal intelligence (7/28) were significantly more than that in symptomatic LGS group (0/22), while the patients with moderate to severe ID (9/28) were significantly less than in the symptomatic LGS group (14/22)

Furthermore, the ID severity was compared between patients with cryptogenic LGS and those with symptomatic LGS (Fig. 1). Significant difference was found between the two groups (*p* = 0.027), which showed that moderate to severe ID was more common in the symptomatic group than that in the cryptogenic group.

### Comparison of ASD and ID between LGS and DS

To explore the relationships among epilepsy, ASD, and ID, we compared psychomotor development abnormalities between patients with LGS and those with DS. The demographic and clinical characteristics of the 45 patients with DS are summarized in Table 3, among whom 37 cases had been reported previously [8]. These patients aged between 2 to 16 years (mean age 8.2 years) at the neurodevelopment evaluation. There was no significant difference in age at the evaluation between the DS group and LGS group (8.2 ± 3.7 vs. 9.3 ± 6.2, mean ± SD, *p* > 0.05). The seizure onset age of patients with DS ranged from 3 days to 18 months, significantly earlier than that of patients with LGS (6.2 ± 3.7 vs. 35.3 ± 29.6, mean ± SD, *p* < 0.05). The most common seizure types were GTCS/secondary GTCS, complex partial seizures (CPS), and myoclonic seizures.

**Table 3:** Demographic and clinical features of patients with Dravet syndrome (*n* = 45)

Gender (male)	32 (71.1%)
Age at conclusion of study (years)	11.3 ± 5.1
Age at evaluation (years)	8.2 ± 3.7
Age at seizure onset (months)	6.2 ± 3.7
Family history of febrile seizures or epilepsy	16 (35.6%)
Antecedent febrile seizures	35 (77.8%)
Seizure type	
GTCS/secondary GTCS	40 (88.9%)
Complex partial	29 (64.4%)
Myoclonic	18 (40.0%)
Atypical absence	15 (33.3%)
Simple partial	9 (20.0%)
Atonic	4 (8.9%)
Tonic	1 (2.2%)
Status epilepticus	28 (62.2%)
EEG characteristics	
Normal	1 (2.2%)
Slow background activity	25 (55.6%)
Only focal discharge	14 (31.1%)
Only generalized discharge	5 (11.1%)
Focal and generalized discharge	25 (55.6%)
MRI abnormality	13 (28.9%)
Treatment with ≥ 3 AEDs	36 (80.0%)
Seizure free	0 (0%)

Value was expressed as *n* (%) or means ± SD

*AEDs* antiepileptic drugs, *GTCS* generalized tonic-clonic seizure

**Fig. 2:**
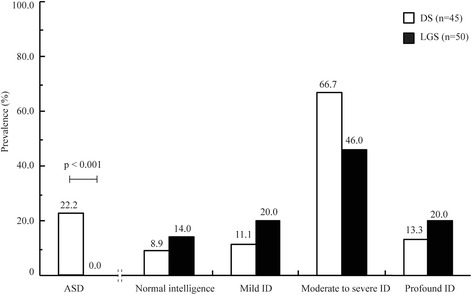
ASD and ID in patients with LGS and those with DS. No ASD was found in the LGS group (0/50), significantly lower than the prevalence of ASD in DS group (10/45, *p* < 0.001), while the cognitive outcome in the two groups shows no significantly difference (*p* = 0.245)

ASD was diagnosed in 22.2% (10/45) of patients with DS, significantly higher than that (0/50) in patients with LGS (Fig. 2, *p* < 0.001). The average ABC scores in the DS patients were 46.8, significantly higher than that in LGS patients (46.8 ± 25.7 vs. 22.1 ± 19.9, mean ± SD, *p* < 0.001). The average CARS scores in DS patients were also significantly higher than that in LGS patients (23.8 ± 7.8 vs. 20.7 ± 6.2, mean ± SD, *p* = 0.036). Among the ten DS patients with ASD, they all had speech delay, narrow interests, and lack of emotional reciprocity; nine of them showed stereotypic behavior; seven of them had short temper; and four of them displayed language regression.

C-WISC and Gesell Developmental Scale were performed on 36 and 9 patients with DS, respectively. According to the IQ or DQ values, 41 patients (91.1%) had ID, including 5 patients (11.1%) with mild ID, 30 (66.7%) with moderate to severe ID, and 6 (13.3%) with profound ID. There was no significant difference in the severity of ID between patients with DS and those with LGS (Fig. 2, *p* = 0.245). Nine of the ten patients with ASD showed ID, among which moderate, severe, and profound ID were found in two, three, and four patients, respectively.

## Discussion

In the present study, no ASD was diagnosed in the 50 patients with LGS, although majority (86%) of them presented ID. The co-morbid prevalence of ASD in LGS was significantly lower than that in DS, but the ID severity did not differ significantly between LGS and DS. These findings suggest that the prevalence of ASD in epilepsy might not be accounted for by the ID. Besides ID, which has been hypothesized as a critical determinant of the co-morbid of epilepsy-ASD-ID triad previously [7, 12], other factors should be considered.

Lennox-Gastaut syndrome is a typical severe epileptic encephalopathy associated with psychomotor developmental abnormalities. Intellectual disability is a prominent feature of LGS. About 20–60% of LGS patients have apparent ID at the onset of seizures, and the proportion of patients with serious ID may increase to 75–95% 5 years after the seizure onset [2, 13]. In this study, majority (86%) of patients with LGS demonstrated ID, among which moderate and severe ID was common.

Previous studies have suggested several risk factors for ID in patients with LGS, such as nonconvulsive status epilepticus, previous diagnosis of infantile spasms, symptomatic etiology, and early seizure onset age [3, 14]. The present study confirmed symptomatic etiology and early seizure onset age as predictors of ID. After follow-up for more than 3 years, 25% of patients with cryptogenic LGS in this cohort still had normal intelligence, whereas all patients with symptomatic LGS showed ID of varying severity. Similar phenomenon was observed in a previous study, 33% of cryptogenic LGS patients had normal or borderline intelligence after a 3-year follow-up, as compared with only 3% of normal intelligence in patients with symptomatic LGS [13]. Previous study has suggested that cortical or cortical-subcortical connection intact is crucial in the cognition development [15]. It is unknown whether the severe ID in patients with symptomatic LGS was due to impaired cortical or cortical-subcortical connection. The seizure onset age is also suggested to be one of the risk factors for intellectual impairment. The seven patients with favorable cognitive outcome had a mean onset age of 50.6 months, while those with ID had a mean onset age of 26.2 months, consistent with a previous study that revealed seizure onset before age 3 years being a significant risk factor for ID in LGS [14].

ASD varies greatly in different epilepsies. The prevalence of ASD was reported to range from 17 to 63% in tuberous sclerosis [16], 24 to 61% in DS [8, 17], and be 35% in infantile spasms [18]. However, the prevalence of co-morbid ASD in LGS remains unclear, although several cases with autism or autistic behavior have been reported [4, 5]. In this study, none of the patients with LGS met the diagnostic criteria of ASD, although three of them exhibited more or less autistic behaviors.

Several variables, such as seizure onset age [19], seizure type [20], ID, and gender [21, 22], were suggested to be risk factors for ASD in epilepsy. ID is one of the factors that should be considered. Patients with epilepsy and ID had an increased risk of ASD (13.8%) relative to those with epilepsy but without ID (2.2%) [23]. A previous study proposed that the prevalence of ASD in epilepsy was accounted for by the degree of ID [7]. The proposal was not supported by the evidence in the present study. The prevalence of ASD in DS was significantly higher than that in LGS, albeit the prevalence and severity of ID were similar in the two forms of epileptic encephalopathy. Factors other than ID should be considered.

Seizure onset age was suggested to be one of the risk predictors for the ASD in the present study. Generally, seizure in DS occurs before the first year of life, while seizure in LGS happens between 1 and 8 years old [9–11]. In this study, the mean onset age in the patients with DS was 6.2 months, whereas that in patients with LGS was 35.3 months. We noticed that the three LGS patients with autistic behaviors suffered their first seizure before 1 year old, which were at 2, 4, and 6 months, respectively. Previous studies have shown that the prevalence of ASD was highest among children whose seizures started before age 2 years [24]. Generally, the stage before age 2 is critical for brain development [25]. Early onset seizure during this stage is possibly an indicator of neurodevelopmental abnormalities, which may account for ASD. However, the seizures are more visible relatively to autistic behaviors, which are often ignored by parents in daily care. It is therefore undetermined when the ASD began in the present LGS and DS cohorts, which is one of the limitations of this study.

Seizure types differed between LGS and DS in this study (Tables 1 and 3), which has been suggested to be one of the possible risk factors for ASD [20, 26]. However, it is unknown whether the seizure type itself or the underlying pathogenesis contributes to ASD.

Current data suggests that genetic etiology plays a critical role in the pathogenesis of epilepsy as well as the co-morbidity. DS is typically a genetic epileptic encephalopathy caused by *SCN1A* mutations in the majority [27]. Our recent study demonstrated 50% of DS patients had *SCN1A* mutations [28]. In contrast, LGS remains elusive in genetic etiology in the majority, although mutations in genes such as *GABRB3* and *ALG13* were occasionally detected [29]. Only a few patients with LGS were found to have causative mutations [28, 30]. Previous studies have suggested a possible involvement of several genes in ASD, such as *SCN1A*, *SCN2A*, *GABRA1*, and *GABRB1* [31]. Additionally, some genetic epilepsies are at high risk of ASD, such as tuberous sclerosis/*TSC2* gene [16] and Fragile X syndrome/*FMR1* gene [32]. However, little is known about the relative contribution of genetic factors in ASD and the underlying mechanisms. Due to the incompleteness of genetic information in the patients in this study, the relationships among genetic risk factors, ASD, and epilepsy warrant further investigation.

## Conclusion

This study showed that the co-morbidity of ASD was significantly lower in LGS than in DS, although these two epileptic encephalopathies had a similar prevalence and severity of ID. It is suggested that other factors, besides ID, would be involved in the pathogenesis of ASD in epilepsy. Further studies with large sample size and basic researches are required to unveil the underlying mechanisms and interaction among epilepsy, ASD, and ID.

## Abbreviations

ABC: Autism Behavior Checklist
AEDs: Antiepileptic drugs
ASD: Autism spectrum disorders
CARS: Childhood Autism Rating Scale
CPS: Complex partial seizures
C-WISC: Chinese Wechsler Intelligence Scale for Children
DQ: Developmental quotient
DS: Dravet syndrome
DSM-5: Diagnostic and Statistical Manual of Mental Disorders, Fifth Edition
EEG: Electroencephalogram
GTCS: Generalized tonic-clonic seizure
ICD-10: International Classification of Diseases, Tenth Edition
ID: Intellectual disability
IQ: Intelligence quotient
LGS: Lennox-Gastaut syndrome
